# Neuroinflammation in Low-Level PM2.5-Exposed Rats Illustrated by PET via an Improved Automated Produced [^18^F]FEPPA: A Feasibility Study

**DOI:** 10.1155/2022/1076444

**Published:** 2022-06-07

**Authors:** Mei-Fang Cheng, Tsun-Jen Cheng, Yue Leon Guo, Ching-Hung Chiu, Hung-Ming Wu, Ruoh-Fang Yen, Ya-Yao Huang, Wen-Sheng Huang, Chyng-Yann Shiue

**Affiliations:** ^1^Department of Nuclear Medicine, National Taiwan University Hospital, Taipei, Taiwan; ^2^Institute of Environmental and Occupational Health Sciences, National Taiwan University, Taipei, Taiwan; ^3^Department of Neurology, Changhua Christian Hospital, Taiwan; ^4^Molecular Imaging Center, National Taiwan University, Taipei, Taiwan; ^5^Institute of Medical Device and Imaging, National Taiwan University College of Medicine, Taipei, Taiwan; ^6^Department of Nuclear Medicine, Taipei Veterans General Hospital, Taipei, Taiwan; ^7^Department of Nuclear Medicine, Taipei Medical University Hospital, Taipei, Taiwan; ^8^Department of Nuclear Medicine, Cheng-Hsin General Hospital, Taipei, Taiwan; ^9^PET Center, Department of Nuclear Medicine, Tri-Service General Hospital, Taiwan

## Abstract

**Background:**

[^18^F]FEPPA is a potent TSPO imaging agent that has been found to be a potential tracer for imaging neuroinflammation. In order to fulfill the demand of this tracer for preclinical and clinical studies, we have developed a one-pot automated synthesis with simplified HPLC purification of this tracer, which was then used for PET imaging of neuroinflammation in fine particulate matter- (PM2.5-) exposed rats.

**Results:**

Using this automated synthesis method, the RCY of the [^18^F]FEPPA was 38 ± 4% (*n* = 17, EOB) in a synthesis time of 83 ± 8 min from EOB. The radiochemical purity and molar activities were greater than 99% and 209 ± 138 GBq/*μ*mol (EOS, *n* = 15), respectively. The quality of the [^18^F]FEPPA synthesized by this method met the U.S. Pharmacopoeia (USP) criteria. The stability test showed that the [^18^F]FEPPA was stable at 21 ± 2°C for up to 4 hr after the end of synthesis (EOS). Moreover, microPET imaging showed that increased tracer activity of [^18^F]FEPPA in the brain of PM2.5-exposed rats (*n* = 6) were higher than that of normal controls (*n* = 6) and regional-specific.

**Conclusions:**

Using the improved semipreparative HPLC purification, [^18^F]FEPPA has been produced in high quantity, high quality, and high reproducibility and, for the first time, used for PET imaging the effects of PM2.5 in the rat brain. It is ready to be used for imaging inflammation in various clinical or preclinical studies, especially for nearby PET centers without cyclotrons.

## 1. Introduction

Neuroinflammation is an inflammatory and adaptive response within the central nervous system (CNS) [1] and is the driving force for disease progression, such as Alzheimer's disease (AD) [2], major depressive disorder [3], schizophrenia [4], and brain injuries [4]. Activated in response to neuropathologies [5, 6], microglia and astrocytes have been known to be the predominant mediators during the neuroinflammatory process. The transmembrane domain protein, translocator protein-18 kDa (TSPO), is variously expressed throughout the body and has low expression within the brain [7–9]. However, TSPO levels are significantly increased in the brain when microglia and astrocyte are activated [10, 11]. Recently, the results of meta-analyses of TSPO levels in mild cognitive impairment and AD further supported the association of increased neuroinflammation during the progression of mild cognitive impairment and AD, relative to healthy controls [12]. In addition to CNS, an increase in TSPO expression has also been seen in a wide variety of malignant human cells and tissues including brain cancers [13, 14], prostate cancers [15, 16], colon cancers [17–19], breast cancers [20, 21], esophageal cancers [22], endometrial carcinomas [23], ovarian cancers, and hepatic carcinomas [24]. Albeit its lack of specificity to activated microglia, the TSPO levels may at times reflect neuroinflammation *in vivo* [10].

Exposure to fine particulate matter (PM2.5) has been linked to adverse neurological and behavioral health effects, including increased risk for cognitive decline (AD) [25, 26], Parkinson's disease [27], ischemic stroke [28], and anxiety or depression [29, 30] in epidemiology studies. Increases in neuroinflammation and oxidative stress have been identified as putative mechanisms by which fine PM2.5 may impair central nervous system function [31, 32], an example of which occurs when exacerbated and unregulated microglial proinflammatory responses play crucial factors involved in brain damage [33]. Chronic activation of the microglia (reactive microgliosis) has been implicated in neuronal injury and neuronal damage after exposure to PM2.5 [34]. One hallmark of microglial activation is the overexpression of TSPO [35, 36]. To the best of our knowledge, the application of a TSPO imaging agent for studying the effects of PM2.5 on human health has not been reported. Thus, we have developed an one-pot automated synthesis of [^18^F]FEPPA, a potent TSPO imaging agent, and used it to investigate (1) whether PET imaging can noninvasively detect microglia activation after chronic subacute ambient PM2.5 exposure in spontaneous hypertensive rats and (2) whether there is a specific pattern of microglia activation in the brain after chronic subacute ambient PM2.5 exposure.

The first TSPO PET ligand, [^11^C]PK-11195 was synthesized more than two decades ago [37–39]. However, its utility was limited due to its low brain penetration, high nonspecific binding, high plasma protein binding, and short half-life (for review, see [37, 38]). Therefore, several second-generation ^18^F-labelled TSPO PET ligands, including [^18^F]FEPPA [40–43], [^18^F]FEDAA1106 [44, 45], [^18^F]DPA-714 [46, 47], and [^18^F]PBR06 [48, 49] have been developed (for review, see [50, 51]). Recently, a novel third-generation TSPO PET ligand, [^18^F]GE180 was developed [52–54] and proved to be useful for TSPO imaging [53, 55, 56]. However, its application for neuroimaging is very limited as it suffers from very low brain penetration, similar to that of [^11^C]PK-11195. Specifically, in humans, the VT of [^18^F]GE180 is 20-fold lower than that of [^11^C]PRB28 [57]. Among these tracers, [^18^F]-N-(2-(2-fluoroethoxy)benzyl)-N-(4-phenoxypyridin-3-yl)acetamide ([^18^F]FEPPA) shows outstanding properties regarding affinity, stability, lipophilicity, and radiosynthesis [43] and has been used in several preclinical [40, 58, 59] and clinical settings [60–66]. In order to facilitate the usefulness of [^18^F]FEPPA in both preclinical and clinical studies, it is imperative to have a fully automated, simple, high yield, and reliable manufacturing process method available for the production of this tracer. Thus, we have adopted Wilson's method [43], with some modifications, to fully automate the synthesis of this potent TSPO imaging agent using a TRACERlab Fx_FN_ module (GE Healthcare, Milwaukee, WI) with ethanol and water for purification in high quality and high reproducibility. Vignal et al. reported a similar synthesis using ethanol, water, and phosphoric acid for purification of [^18^F]FEPPA [42]. We report herein (1) a one-pot automated synthesis of [^18^F]FEPPA with a simplified HPLC purification, (2) the USP compliant QC and stability tests of [^18^F]FEPPA, and (3) microPET imaging of [^18^F]FEPPA in PM2.5-exposed rats.

## 2. Materials and Methods

The precursor (N-[[2-[2-[[(4-methylphenyl)sulfonyl]oxy]ethoxy]phenyl]methyl]-N-(4-phenoxy-3-pyridinyl) acetamide, TsEPPA, 1) and the nonradioactive authentic sample N-(2-(2-fluoroethoxy)benzyl)-N-(4-phenoxypyridin-3-yl) acetamide (FEPPA, 2) were purchased from ABX Advanced Biochemicals (Radeberg, Germany). All other chemicals and solvents were purchased from either Sigma-Aldrich (Milwaukee, WI, USA) or Acros Organics (Morris Plains, NJ, USA) and used without further purification. [^18^O]O_2_H (>98% enriched) was purchased from Rotem Industries (Beer Sheva, Israel). Aqueous [^18^F]Fluoride was produced in our PET Trace cyclotron (GE Medical Systems, Uppsala, Sweden) via ^18^O(p, n)^18^F nuclear reaction. All Sep-Pak® cartridges were purchased from Waters Associates (Milford, MA, USA).

### 2.1. Automated Radiosynthesis

**Scheme 1:**
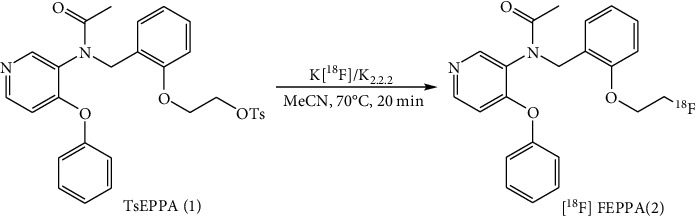
Radiosynthesis of the [^18^F]FEPPA (2).

The [^18^F]FEPPA (2) was produced by automated synthesis via fluorination of the TsEPPA (1) with K[^18^F]/K_2.2.2_ followed by purification with HPLC, to give [^18^F]FEPPA (2) as previously reported (Scheme 1) [43].

**Figure 1:**
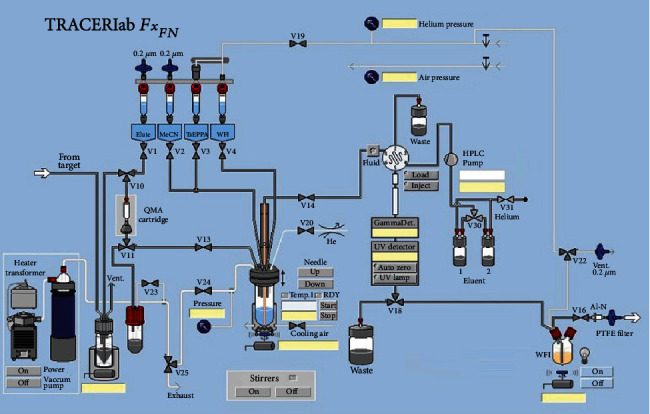
Modified TRACERLab Fx_FN_ module for the [^18^F]FEPPA (2) radiosynthesis.

The [^18^F]FEPPA (2) was produced by automated synthesis using a modified TRACERlab Fx_FN_ module (GE Healthcare, Milwaukee, WI; Figure 1). Briefly, fluorination of TsEPPA (1) with K[^18^F]/K_2.2.2_ in anhydrous MeCN at 70°C for 20 min gave the crude product (1) (Scheme 1). After dilution with H_2_O, the crude product (1) was purified with a semipreparative HPLC (Waters Xterra RP-18, 10 *μ*m, 10 × 250 mm, 35% aqueous ethanol in water for injection, 254 nm, 4 mL/min). The fraction containing [^18^F]FEPPA (2) was collected, reformulation, and sterile filtration to provide (2) with <10% of ethanol concentration for PET imaging of the effects for PM2.5 in rats. Please refer to Supplementary data for detailed steps of radiosynthesis.

### 2.2. Quality Control (QC) and Stability Tests of the [^18^F]FEPPA (2)

The radiochemical purity, chemical purity, and molar activity of 2 were analyzed with an HPLC system (Agilent 1100 series) equipped with a Bioscan FC3300 flow count radioactivity detector (2^″^ × 2^″^ pinhole) and a UV detector (254 nm) using a Waters Xterra column (RP-18, 5 *μ*m, 4.6 × 250 mm) with 50% aqueous MeCN as the eluent and a flow rate of 1.0 mL/min. The chemical identity of the [^18^F]FEPPA (2) was confirmed by coinjection with a nonradioactive authentic FEPPA. The radiochemical impurity of 2 was further analyzed with radio TLC (Silica gel 60 F254 plate, 10 cm, ethyl acetate/hexane (3/1)) and detected with a Raytest miniGita radio-TLC scanner (Raytest, Straubenhardt, Germany). Please refer to Supplementary data and Figure S1 for details.

Other items of QC test and corresponding criteria of 2 were set according to the U.S. Pharmacopoeia (USP) for radiopharmaceuticals [67], which included visual inspection, pH, half-life of radionuclide, radionuclidic purity, radiochemical purity, chemical purity, residual K_2.2.2_, residual solvents, bacterial endotoxins, filter integrity, and sterility test.

The stability of 2 at 21 ± 2°Cwas monitored with both TLC and HPLC as described above for up to 4 hr after EOS.

### 2.3. MicroPET Imaging of the [^18^F]FEPPA (2) Injection in Rats Exposed to Ambient Fine Particulate Matter (PM2.5)

#### 2.3.1. Animals

Male spontaneously hypertensive rats (7-week-old, average weight of 350 g, *n* = 12) were obtained from the National Laboratory Animal Center (Taipei, Taiwan) and were randomly classified into ambient fine PM exposure group (aerodynamic diameter of <2.5 *μ*m, PM2.5; *n* = 6) and high-efficiency particulate air- (HEPA-)-filtered air (FL, *n* = 6) conditions group using Taipei Air Pollution Exposure System (TAPES) for health effects [68, 69] for 24 hours per day, 7 days per week, for a total of 6 months. The coarse (PM2.5-10) and fine (PM2.5) size concentrations constituted 0.4 and 99.6% of the whole-body exposure system [70]. Therefore, the rats were mostly exposed to PM2.5. The rats were housed in ventilated cages under a conditioned environment as illustrated in our previous studies [68]. Lab diet and water were provided *ad libitum* during the study. The animal experiments adhered to protocols of the Institutional Animal Care and Use Committee (IACUC) of the Laboratory Animal Center at National Taiwan University (ACUC no: 20160025).

At the end of exposure, [^18^F]FEPPA microPET/CT brain scan was performed for each rat to assess the degree of neuroinflammation. During scanning, rats were anesthetized by passive inhalation of a mixture of isoflurane (5% for induction and 2% for maintenance) in oxygen, followed by a bolus tail vein injection of 24 ± 10 MBq, *n* = 6) of (2). Brain transmission and emission scans of rats were acquired with a small animal Argus PET/CT scanner (SEDECAL, Madrid, Spain). Ten minutes posttail vein injection of (2), static sinograms were produced for 30 min for each organ of interest. Images were analyzed using commercial PMOD software (PMOD Technologies LLC, version 3.6, Zurich, Switzerland) after coregistration the microCT images to the innate MR atlas. Regional brain radioactivity concentrations (MBq/cm^3^) were normalized to the injected activity (MBq) and the weight (g) of organ to yield a semiquantitative measurement of radiotracer binding, standardized uptake value (SUV) [71]. The mean SUV of each brain region (volume of interest, VOI) such as the whole brain, frontal lobe, parietal lobe, insular lobe, occipital lobe, midbrain, temporal lobe, hippocampus, retrosplenial cortex in the temporal lobes, and cerebellum were calculated and analyzed.

### 2.4. Immunohistochemical Analysis

After PET image acquisition, all rats were sacrificed. The brain tissues were processed using an automated tissue processor (Shandon Excelsior, Thermo Scientific, UK), embedded in paraffin, and cut at a thickness of 3-5 *μ*m for immunohistochemical (IHC) staining. Ionized calcium binding adaptor molecule (Iba1) is a macrophage-specific calcium-binding protein, participating in membrane ruffling and phagocytosis in the activated microglia. For Iba1 staining, brain tissues were stained against the activated microglial marker Iba1 (GeneTex, San Antonio, TX, USA) [72]. The percentage of areas occupied by the stained nuclei of activated microglia at 40x high power field (0.26 mm/pixel) in the hippocampal region of the rats was calculated using a digital slide scanner (MoticEasyScan, Motic®, Canada). The brain tissues were examined by a histopathologist blinded to the exposure data.

### 2.5. Statistical Analysis

Data was expressed as mean ± standard deviation (SD). Wilcoxon rank sum test was used to compare different regions of brain in PM2.5 and FL rats. *P* < 0.05 was regarded as statistically significant. Statistical analyses were performed using JMP®, version 10 statistical software package (SAS Institute Inc., Cary, NC, USA).

## 3. Results

### 3.1. A One-Pot Automated Synthesis of the [^18^F]FEPPA (2) Using a Modified Fx_FN_ Module

In this paper, we presented the detailed automated production of [^18^F]FEPPA along with a full set of quality control specifications and results in USP compliance. Using this modified purification method, we were able to routinely produce 2 with a Fx_FN_ module in 38 ± 4% yield (EOB, *n* = 17) in a synthesis time of 83 ± 8 min from EOB. Typically, starting with 35 GBq of [^18^F]Fluoride, 8 GBq of 2 was produced at EOS. Both the chemical and radiochemical purities of 2 were greater than 90% with a molar activity of 209 ± 138 GBq/*μ*mol (*n* = 15, EOS) and were used for PET imaging of the effects of PM2.5 in rats.

The QC test results of 2 are tabulated in Table 1. Moreover, the stability test showed that 2 synthesized by this method was stable at room temperature (21 ± 2°C) for up to 4 hr after EOS (Table 2). Detailed QC data for [^18^F]FEPPA produced using our method disclosed herein are shown in supplementary data.

### 3.2. MicroPET Imaging of the [^18^F]FEPPA (2) Injection in Rats Exposed to Ambient Fine Particulate Matter (PM2.5)

The mean mass concentration of PM2.5 during the exposure period was 10.8 ± 3.8 *μ*g/m^3^. The predominant chemical composition of the trapped particles in the PM group was sulfur (16.0% of mean PM2.5 concentration), potassium (1.2%), and iron (0.6%), similar to that in previous PM exposure data [68, 70]. There was no significant alterations in the weight of the rats after ambient PM2.5 exposure between the PM2.5 and FL groups.

**Figure 2:**
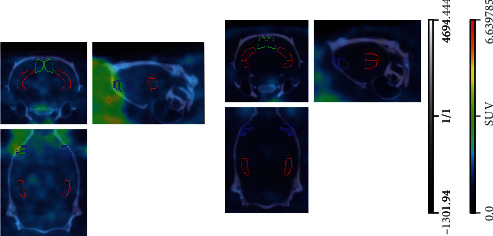
Representative imaging of coronal, sagittal, and axial sections of rat brain exposed to ambient PM2.5 ((a) PM2.5, left panel) versus filtered air ((b) FL, right panel).

**Figure 3:**
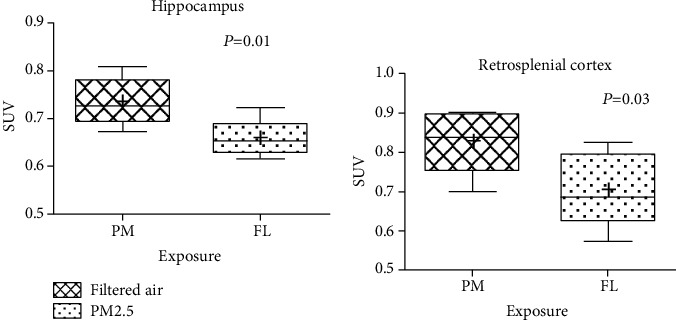
Tracer distribution of the [^18^F]FEPPA (2) in the hippocampus (a) and retrosplenial region (b) of the temporal lobes.

Typical whole-brain biodistribution of [^18^F]FEPPA in the PM2.5 and FL rats was depicted in Figure 2. Increased tracer activity of [^18^F]FEPPA in selected brain regions was shown in Table 3 and Figure 3 and expressed as SUV mean.

Specifically, significant increased [^18^F]FEPPA tracer activity was observed in the temporal lobe of the PM2.5 compared to that of the FL rats (*P* = 0.04), especially in the hippocampus (Figure 2, red contour, *P* = 0.01) and retrosplenic region (Figure 2, green contour, *P* = 0.03). A trend of increased tracer activity was found at insular areas (Figure 2, indigo contour) and frontal lobe (not shown) in the PM2.5 rats, but not reaching statistical difference (Table 3).

### 3.3. Immunohistochemical (IHC) Staining of the Rat Brain

**Figure 4:**
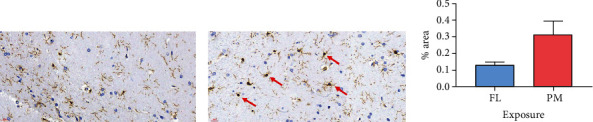
The immunostaining (brown-colored nuclei, red arrows) in the hippocampus of the PM rats (a), FL rats (b), and the differences between these two groups (c).

The IHC staining of the hippocampus showed that the % of area with Iba1 staining at 40x high power field were 0.130 ± 0.017% in the FL group vs. 0.313 ± 0.081% in the PM2.5 group (Figure 4, *P* = 0.05).

There is a trend for higher Iba1 staining (brown-colored nuclei, red arrows) in the hippocampus of PM rats (a) compared to the FL group (b), but the difference is not statistically significant (staining area of 0.313 ± 0.081% vs. 0.130 ± 0.017%, *P* = 0.05 (c)).

## 4. Discussion

[^18^F]FEPPA is a potent TSPO imaging agent that has been found to be a potential tracer for imaging neuroinflammation. In order to fulfill the demand of this tracer for preclinical and clinical studies, we have developed a one-pot automated synthesis with simplified HPLC purification of this tracer, which was then, for the first time used for PET imaging of the effects of PM2.5 in rats.

[^18^ F]FEPPA (2) has been synthesized by several methods in various radiochemical yields and radiochemical purities [42, 43, 58, 73]. Initially, 2 was synthesized manually by nucleophilic fluorination of the corresponding tosylate-precursor (TsEPPA, 1) with [^18^F]Fluoride, followed by purification with HPLC, to give 2 in 71~85% yield (EOB) (Scheme 1) [43]. Later, several automated syntheses of 2 were reported [42, 58, 73] (Table 4).

**Figure 5:**
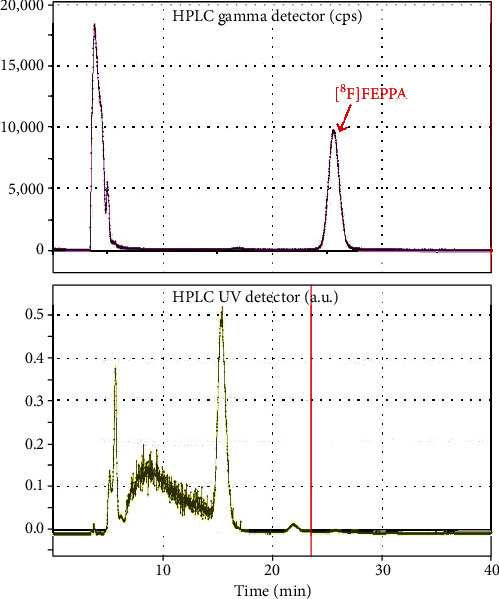
Representative semipreparative HPLC purification chromatogram of the [^18^F]FEPPA (2).

However, most of these automated syntheses used toxic MeCN as the solvent for semipreparative HPLC purification, and thus, reformulation was necessary. As a result, we and others [42] have chosen to use aqueous ethanol as the mobile phase for purification of 2 with HPLC. The HPLC purification conditions of 2 have been optimized by using a Waters Xterra RP-18 column (10 *μ*m, 10 × 25 mm) and eluting with different flow rates and different concentrations of aqueous ethanol as mobile phase (Figure S2). Although the retention time was slightly longer when separation was done using 35% instead of 40% EtOH, but because of practical considerations, including nonradioactive impurity separation and easy-to-prepare mobile phase (Figure S3), 35% EtOH at a flow rate of 4 mL/min was chosen as the best condition for purification 2 (Figure 5).

However, a small amount of [^18^F]Fluoride was detected in the eluate, which may have had an impact on PET image quality [74]. Thus, the eluate was further passed through an Alumina-N cartridge and a PTFE sterile filter in series to remove the residual [^18^F]Fluoride and resulted in the pure and sterile 2 in 38 ± 4% yield (EOB, *n* = 17) in a synthesis time of 83 ± 8 min from EOB, which is comparable to 2 synthesized by other methods (Table 4). For TSPO-targeting studies, the molar activity is recommended as high as possible as required for studies targeting receptors. Compared to previous published methods, our method achieved a reasonable and high molar activity of 209 ± 138 GBq/*μ*mol, EOS (*n* = 17) as shown in Table 4. Quality control and stability tests confirmed that 2 synthesized by this method met the USP. As far as we know, our method is still the only to address residual [^18^F]Fluoride concern during automated production, as well as the QC item of residual [^18^F]Fluoride determination using radio-TLC analysis, in order to achieve high quality of produced [^18^F]FEPPA for preclinical or clinical studies.

PET imaging in rats showed that the whole-brain [^18^F]FEPPA tracer activity of the PM2.5 rats was significantly higher than that of their age-matched FL rats. In addition, the brain tracer activity of [^18^F]FEPPA was found to be regional-specific, and the difference was more pronounced in the temporal and insular lobes. In the temporal lobe, only the hippocampus and retrosplenial cortex showed statistical higher [^18^F]FEPPA activity in the PM2.5 rats when compared to that of the FL rats (Table 3, Figure 3). A trend of increased tracer activity was found at both insular and frontal regions in the PM2.5 rats but did not reach a statistically significant difference.

The IHC staining results showed that although there was not a statistically significant difference in the hippocampus, a trend of increased Iba1 staining in the microglia cells was observed in the PM2.5 group compared to that of the filtered air group, which suggested that microglial activation and inflammation, especially in the temporal lobe, may play important roles in the response of the brain to traffic-related PM.

Taken collectively, we have developed an improved method to automatically produce [^18^F]FEPPA in high quantity, high quality, and high reproducibility, and for the first time using it to noninvasively elucidate the microglial changes in different brain regions of rats after subchronic real-world exposure to ambient PM2.5. Our study confirmed that chronic subacute ambient PM2.5 exposure can lead to diffuse microglial activation. The brain area that was especially vulnerable to PM2.5 effects was the temporal lobe, particularly the hippocampus and retrosplenial cortex. These regions played crucial roles in memory, learning, and navigation, especially in the hippocampus. However, there are several limitations of the present rodent study. First, the limited sample size may influence the overall power of the test. On the other hands, this also suggests that with larger sample sizes, perhaps more brain regions will be affected, as we did observe a trend of increased [^18^F]FEPPA activity (not reaching statistical significance) in the insular and frontal regions of PM2.5 rats compared to their controls. Second, hypertensive rats were used in the current experiment, a subgroup that may be more vulnerable to fine PM exposure than normotensive rats. Spontaneous hypertensive rats were chosen since cardiovascular diseases are recognized as risk factors for developing neurodegenerative disease in humans and therefore may be more susceptible to traffic-related PM2.5 exposure. Third, blocking experiment using a nonradioactive FEPPA or other nonradioactive TSPO ligand was not performed in the current study. Nonetheless, [^18^F]FEPPA is a relatively popular ligand that had been used in many preclinical and clinical studies [75, 76], including and not limited to psychiatric disorders [62, 77]. Various studies have also demonstrated that [^18^F]FEPPA reliably binds to TSPO in the brain [78–80]. Lastly, not all regions of the brain were analyzed for Iba1 staining. Nevertheless, the areas of increased Iba1 staining were consistent with the [^18^F]FEPPA PET findings.

## 5. Conclusions

With this one-pot automated synthesis and improved purification of the [^18^F]FEPPA (2) using a Fx_FN_ Module, [^18^F]FEPPA (2) can be produced with high quality, quantity, and reproducibility. PET imaging in rats exposed to low-level PM2.5 demonstrated that pulmonary exposure to fine PM may exert health effects on specific areas of the brain, including the hippocampus. The microglial activation and inflammation can be noninvasively evaluated and followed by [^18^F]FEPPA PET imaging. The role of microglial response after low-level fine PM2.5 exposure warrants further investigation in humans. The applications of [^18^F]FEPPA (2) for studying inflammation in the peripheral organs of animals and humans are in progress.

## Figures and Tables

**Table 1:** The QC tests of the [^18^F]FEPPA (2).

Items tested	Acceptance criteria			
Visual inspection	Clear, colorless solution	Pass	Pass	Pass
pH value	5-8	7	7	7
Residual K_2.2.2_ (*μ*g/mL)	<50	<50	<50	<50
Radionuclidic purity (%)	>99.5	99.93	99.96	99.62
Half-life (min)	110 ± 5	108	115	115
Radiochemical identity (min)	∣R_t_-R_t_ (reference)∣≤0.5	0.02	0.00	0.07
Radiochemical purity (%)	>90	99.9	99.8	99.8
Residual solvent analysis	EtOH < 10% Acetone < 0.5% MeCN < 0.04%	4.6849% 0.1454% 0.0100%	0.1673% 0.0324% 0.0014%	2.2298% 0.0010% 0.0008%
Residual [^18^F]Fluoride (%)	<5	0.01	0.02	0.59
Bacterial endotoxins (EU/V)	<175	<17.5	<17.5	<17.5
Filter integrity test (psi)	>45	48.5	46.6	46.0
Sterility test	Sterile	Sterile	Sterile	Sterile

**Table 2:** Stability of three consecutive productions of the [^18^F]FEPPA (2) (*n* = 3).

Items tested	Acceptance criteria	Elapsed time (hr)	Run 1	Run 2	Run 3
Radiochemical purity (%)	>90%	0	99.9	99.8	99.8
		2	99.0	99.3	98.6
		4	98.9	99.4	97.7
Residual [^18^F]Fluoride (%)	<5%	0	0.01	0.02	0.59
		2	0.34	0.73	1.01
		4	1.04	0.23	1.89

**Table 3:** MicroPET Imaging of the [^18^F]FEPPA (2) in ambient PM2.5-exposed (*n* = 6) versus filtered air-exposed (*n* = 6) rats.

Region	PM (SUV mean)	FA (SUV mean)	*P* value
Whole brain	0.85 ± 0.02	0.78 ± 0.03	0.03^∗^
Frontal lobe	0.92 ± 0.02	0.87 ± 0.02	0.047
Parietal lobe	0.73 ± 0.04	0.75 ± 0.03	NS
Insular lobe	0.93 ± 0.04	0.83 ± 0.02	0.047
Occipital lobe	0.64 ± 0.04	0.68 ± 0.03	NS
Midbrain	0.80 ± 0.04	0.81 ± 0.04	NS
Olfactory bulb	0.99 ± 0.04	1.06 ± 0.06	NS
Temporal lobe	0.78 ± 0.02	0.71 ± 0.03	0.04^∗^
Hippocampus	0.73 ± 0.02	0.66 ± 0.02	0.01^∗^
Retrosplenial cortex	0.83 ± 0.04	0.71 ± 0.04	0.03^∗^
Cerebellum	0.85 ± 0.04	0.86 ± 0.05	NS

Abbreviations: FA: filtered air; NS: not significant; PM: particulate matter.

**Table 4:** Synthesis of the [^18^F]FEPPA (2) via various methods.

References	Radiofluorination condition				RCY (%, EOB)	Synthesis time (min)	Number of production	Molar activity (GBq/*μ*mol)	Automate synthesizer	QC (EP or USP)	TLC
	Precursor weight (mg)	Solvent (mL)	Temperature (°C)	Time (min)							
Wilson et al. [43]	5	MeCN 0.7 mL	90	10	71–85	40–50	—	44~100	—	—	—
Vasdev et al. [58]	5	MeCN 0.75 mL	90	10	38 ± 10	36 ± 1	54	222 ± 148	Fx_FN_	—	—
Berroterán-Infante et al. [73]	3.125	MeCN 0.5 mL	90	10	38 ± 3	30	15	241 ± 13	Nuclear Interface	EP	Y^∗^
Vignal et al. [42]	5	MeCN 1 mL	90	10	48 ± 3	55	17	198 ± 125	AllInOne®	—	—
Zammit et al. [59]	2	MeCN 0.4 mL	90	10	41 ± 8	78 ± 4	—	751 ± 163	CPCU	—	—
Chang et al. [74]	5	MeCN 0.6 mL	90	10	50	80	8	149~217	Eckert-Ziegler modular system	—	—
This study	5	MeCN 1 mL	70	20	38 ± 4	83 ± 8	17	209 ± 138	Fx_FN_	USP	Y

^∗^No TLC data were provided.

## Data Availability

The datasets used and/or analyzed during the current study are available from the corresponding authors on reasonable request.

## Supplementary Materials

Supplementary Materials Information on the radiosynthesis and quality control tests are provided.
