# Supplementary material for: Neuroinflammation in Low-Level PM2.5-Exposed Rats Illustrated by PET via an Improved Automated Produced [18F]FEPPA: A Feasibility Study
Source: Mol Imaging. 2022 Jun 7;2022:1076444. doi: 10.1155/2022/1076444 (PMC9328187; doi:10.1155/2022/1076444)

SUPPLEMENTARY DATA

Neuroinflammation in Low-level PM2.5 Exposed Rats Illustrated by PET via An Improved Automated Produced [^{18}F]FEPPA – A Feasibility Study

Mei-Fang Cheng[1,2], Tsun-Jen Cheng[2], Yue Leon Guo[2], Ching-Hung Chiu[1], Hung-Ming Wu[3], Ruoh-Fang Yen[1,4], Ya-Yao Huang*[1, 4-5], Wen-Sheng Huang*[6-8], Chyng-Yann Shiue* [1,4,9]

[1]Department of Nuclear Medicine, National Taiwan University Hospital, Taipei, Taiwan

[2]Institute of Environmental and Occupational Health Sciences, National Taiwan University, Taipei, Taiwan

[3]Department of Neurology, Changhua Christian Hospital, Taiwan

[4]Molecular Imaging Center, National Taiwan University, Taipei, Taiwan

[5]Institute of Medical Device and Imaging, National Taiwan University College of Medicine, Taipei, Taiwan

[6]Department of Nuclear Medicine, Taipei Veterans General Hospital, Taipei, Taiwan

[7]Department of Nuclear Medicine, Taipei Medical University Hospital, Taipei, Taiwan

[8]Department of Nuclear Medicine, Cheng-Hsin General Hospital, Taipei, Taiwan

[9]PET Center, Department of Nuclear Medicine, Tri-Service General Hospital, Taiwan

Automated radiosynthesis

The synthesis was carried out with a modified TRACERlab Fx_{FN} module (GE Healthcare, Milwaukee, WI) in which V14 was connected to a fluid sensor for direct injection of reaction mixture to semi-preparative HPLC, V18 was connected to a product vial for direct transfer of collected product to the product vial, and V16 was connected to a Sep-Pak® Alumina-N Plus Long cartridge and 0.22 μm PTFE sterilizing filter (Millipore, Madrid, Spain) in series to a sterile vial.

Prior to delivery of $[^{18}F]$Fluoride to the modified TRACERLab Fx_{FN} module, each vial was filled with the appropriate solvent and reagent**.**

Vial 1: K_2CO_3 (4.7 mg) in 0.2 mL of water and Kryptofix $_{2.2.2}$ ($K_{2.2.2}$, 14.7 mg) in 0.2 mL of acetonitrile (MeCN).

Vial 2: MeCN (1.0 mL).

Vial 3: Precursor (**1,** 5 mg) in 1.0 mL of anhydrous MeCN.

Vial 4: Water for injection (2.5 mL).

Product vial: Water for injection (1mL).

The automated synthesis module was operated in the following sequences:

1. At the end of bombardment, aqueous $[^{18}F]$Fluoride in 1.4 mL of $[^{18}O]O_2H$, produced in our PET Trace cyclotron (GE Medical Systems, Uppsala, Sweden) via an $^{18}O(p, n)^{18}F$ nuclear reaction, was transferred from the target into a $[^{18}O]O_2H$ collection vial with helium purge.

2. Opened V10 and V23. Aqueous $[^{18}F]$Fluoride was passed through a Sep-Pak® QMA Plus Light cartridge and V11 under vacuum. $[^{18}O]O_2H$ was recovered in the $[^{18}O]O_2H$ recovery vial and the $[^{18}F]$Fluoride was trapped on a Sep-Pak® QMA Plus Light cartridge.
3. Opened V1, V13, and V24. $K_2CO_3/K_{2.2.2}$ solution in Vial 1 was passed through V1, V10, QMA, V11, and V13. $[^{18}F]$Fluoride was collected in Reaction Vessel.
4. Closed V1, V13 and V14. Opened V20 and V24, and evaporated the solution in Reaction Vessel at 110^0C for 2.5 min under a stream of helium from V20.
5. Flushed the solution in Vial 2 into the Reaction Vessel with helium. Closed V2, V13, and V14. Opened V20 and V24, and performed azeotropic distillation of the remaining H_2O at 110^0C for 2.5 min with a stream of helium from V20.
6. Cooled the Reaction Vessel to 40°C and then flushed the solution in Vial 3 into the Reaction Vessel with helium. Closed V3, V13, V14, V20, and V24. Heated the solution at 70°C for 20 min.
7. Flushed the solution in Vial 4 into the Reaction Vessel with helium. Closed V4, V13, V14, V20, and V24. Stirred the solution at 30°C for 15 sec.
8. Opened V14. Injected the reaction mixture in the Reaction Vessel into semi-preparative HPLC (Waters Xterra RP-18, 10 μm, 10×250 mm, 35% aqueous ethanol in water for injection, 4 mL/min) with a stream of helium from V20.

9. At about 25 min post-injection, the $[^{18}F]$FEPPA peak was collected for about

2 min and the solution was transferred through V18 into the product vial, which contained 1 mL water for injection.

10. Opened V16 and flushed the [^{18}F]FEPPA solution in the product vial through an Alumina-N cartridge and a 0.2 μm PTFE sterile filter into a sterile multi-injection vial with a stream of helium from V22.
11. The [^{18}F]FEPPA solution was diluted with saline to provide the [^{18}F]FEPPA (**2**) with <10% of EtOH concentration for PET imaging of the effects of PM2.5 in rats.

Quality Control (QC) and Stability Tests of the [^{18}F]FEPPA (2)

Other items of QC test and corresponding criteria of **2** were set based on the U.S. Pharmacopoeia (USP) for radiopharmaceuticals[73], which included visual inspection, pH, half-life of radionuclide, radionuclidic purity, radiochemical purity, chemical purity, residual $K_{2.2.2}$, residual solvents, bacterial endotoxins, filter integrity, and sterility test.

The radioactivity was determined using a calibrated ion chamber (CRC-15R, Capintec Inc, USA). The radionuclidic purity was determined using a gamma spectrometry system (3 x 3 in. NaI detector) and a multi-channel analyzer (Raytest, Straubenhardt, Germany).

The residual organic solvents in **2** were analyzed by GC equipped with a flame ionization detector (FID) and an Agilent Column (HP Fast Residual Solvent chromatography column (30 m×0.53 mm×1μm). Nitrogen was used as a carrier gas with a flow rate of 2 mL/min. The split/splitless injector was set at 20:1. The initial oven temperature was kept at 40℃ for 1 min followed by an increase of temperature at 14℃/min to 110℃ and then at 30℃/min to 125℃. The oven

temperature was kept at 125℃for 2.5 min, followed by an increase of temperature at 47.5℃/min to 220^{0}C and kept at that temperature for 4 min. Hydrogen and air, at 35 and 250 mL/min, respectively, were used in the GC/FID, with nitrogen (25 mL/min) as a make-up gas. The software, Gina Star (Raytest, Straubenhardt, Germany), was used for data acquisition.

Measurement of bacterial endotoxins was carried out using a Limulus amoebocyte lysate (LAL) test and a sterility test was performed with the proven method meeting the requirement of USP.

The quality of **2** synthesized by this method met the USP criteria. The appearance of **2** was clear, the HPLC retention time of **2** was approximately 6.5 min, the radiochemical purity of **2** was greater than 95% (**Figure S1A**), and the concentration of residual solvents in **2** were EtOH＜10%, Acetone＜0.5%, and MeCN < 0.04% (**Table 1**). The residual [^{18}F]Fluoride in **2** was also assessed by radio-TLC. The retardation factor (R_f) values of [^{18}F]Fluoride and **2** were approximately 0.0 and 0.4, respectively (**Figure S1B**), and the concentration of [^{18}F]Fluoride in **2** was less than 5%. The chemical identity of [^{18}F]FEPPA (**2**) was confirmed by co-injection with a non-radioactive authentic FEPPA (**Figure S1A**). The retention time (R_t) of **2** was 6.7 min**.**

Figure S1. Representative analytical HPLC (A) and radio-TLC chromatograms (B) of the [^{18}F]FEPPA (**2**).

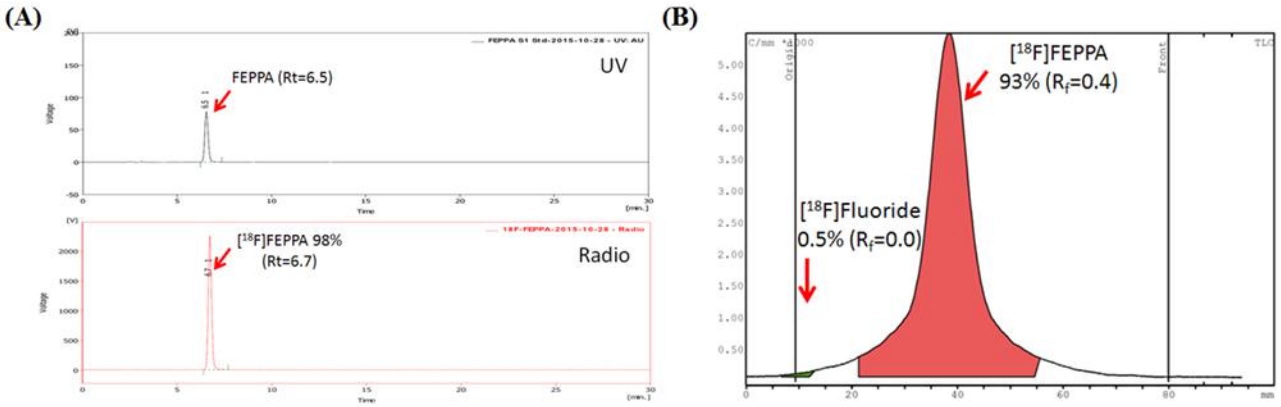

Method Development of semi-preparative HPLC purification of the [^{18}F]FEPPA (2) in this study

The HPLC purification conditions of **2** have been optimized by using a Waters Xterra RP-18 column (10 µm, 10 x 25 mm) and eluting with different flow rates (2~4 ml/min) and different concentrations (35~50%) of aqueous ethanol as mobile phase.

Figure S2. Condition optimization for semi-preparative HPLC purification of the [^{18}F]FEPPA (**2**) (Waters Xterra RP-18, 10 µm, 10×250 mm) using same flow rate (4 mL/min) but different concentrations of aqueous ethanol as mobile phase .

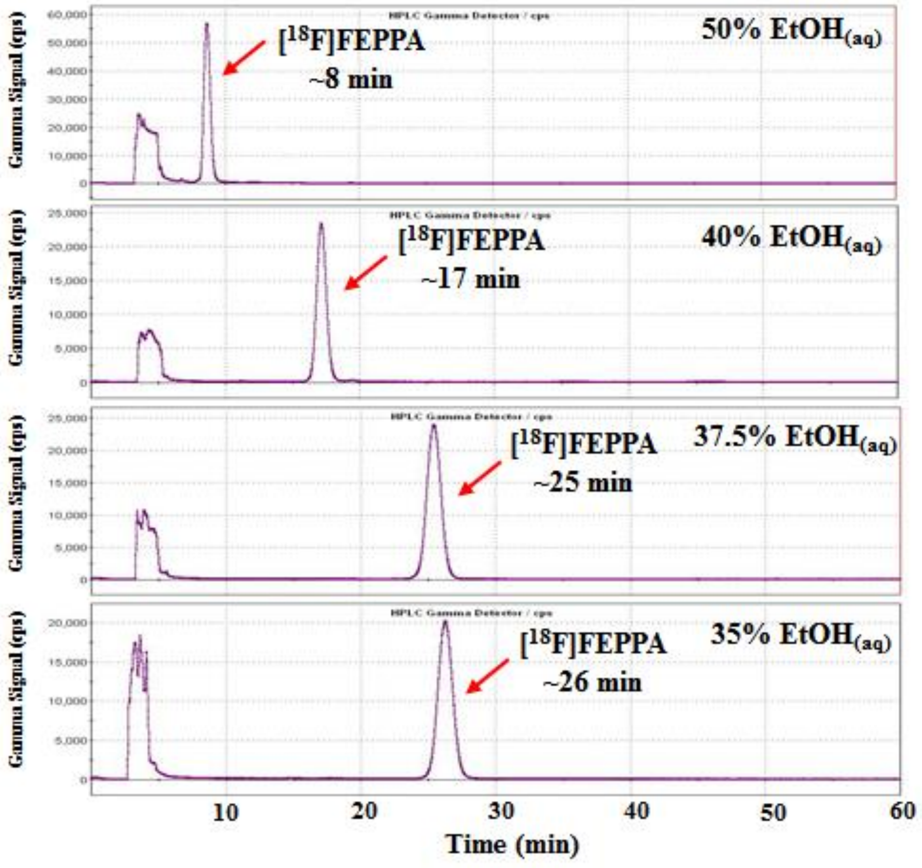

Figure S3. Representative semi-preparative HPLC purification chromatogram of the [^{18}F]FEPPA (**2**) when 40% EtOH of mobile phase was used in semi-preparative HPLC purification.

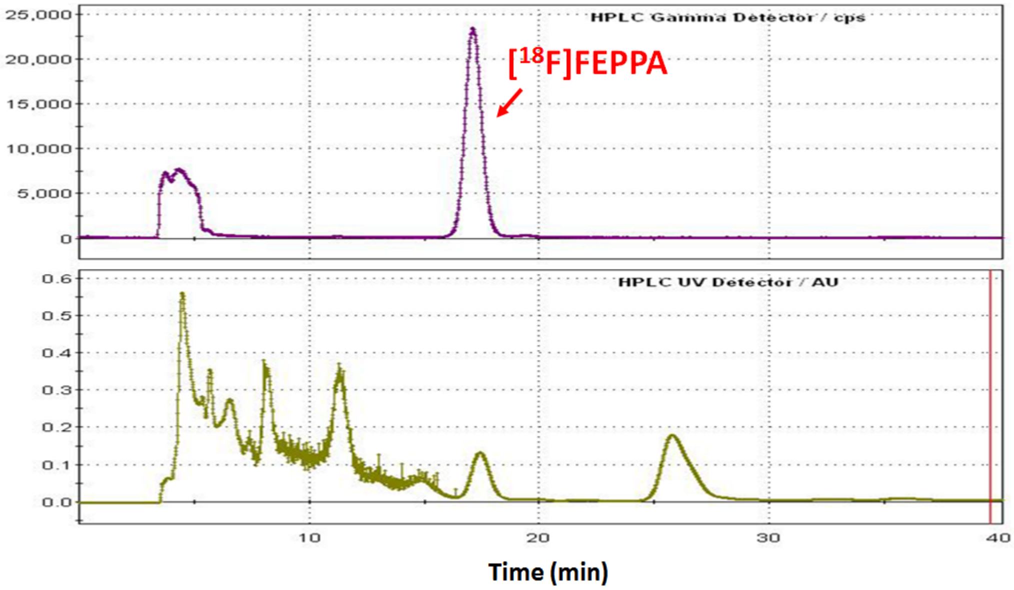

Supplement: Supplementary Materials — Information on the radiosynthesis and quality control tests are provided. [file 1076444.f1.pdf]
